# Endothelial Microparticles Act as Novel Diagnostic and Therapeutic Biomarkers of Diabetes and Its Complications: A Literature Review

**DOI:** 10.1155/2016/9802026

**Published:** 2016-10-10

**Authors:** Fan Deng, Shuang Wang, Liangqing Zhang

**Affiliations:** ^1^Guangdong Medical University, Zhanjiang, Guangdong 524001, China; ^2^Department of Anesthesiology, Affiliated Hospital of Guangdong Medical University, Zhanjiang, Guangdong 524001, China

## Abstract

Diabetes mellitus- (DM-) related vascular diseases attract increased attention due to their high morbidity and mortality. The incidence of obesity, atherosclerosis, coronary heart disease, hypertension, and dyslipidemia is significantly higher in DM patients, with an earlier onset and faster progression compared with non-DM patients. DM-related vascular diseases including macrovascular and microvascular complications are characterized by endothelial dysfunction. Therefore, a better understanding of the etiology and mechanisms of endothelial dysfunction is important for the diagnosis and treatment of DM. Endothelial microparticles (EMPs) are new diagnostic and therapeutic targets and biomarkers in DM-related vascular disease. Circulating EMPs containing biologically active substances act as intercellular signals under physiological and pathological conditions. They serve as biological markers of altered vascular endothelium and reflect the pathological progression and diminished endothelial function of blood vessels. Recent evidence suggests that the plasma level of EMPs is significantly higher in DM patients than in healthy population and is significantly correlated with DM-related complications. These observations have prompted speculation that EMPs play a crucial role in the pathophysiology of DM. This review summarizes the known and potential roles of EMPs in the diagnosis, staging, treatment, and clinical prognosis of DM and related vascular diseases.

## 1. Introduction

Diabetes mellitus (DM) is a metabolic disease characterized by chronic hyperglycemia and long-term disturbances in carbohydrate, lipid, and protein metabolism inducing chronic progressive dysfunction and failure of visual, nervous, renal, cardiac, and blood vascular systems [1]. The incidence of DM is progressively increasing in both developed and developing countries [2]. As of 2014, an estimated 387 million individuals worldwide have been diagnosed with DM and the prevalence is expected to rise to 592 million by 2035 [3]. In addition, the incidence of obesity [4], atherosclerosis [5], coronary heart disease (CAD) [6], hypertension [7, 8], and dyslipidemia [9] is significantly higher in DM patients, with an earlier onset and faster progression compared with non-DM patients [10]. All of these pathological conditions are closely related to vascular diseases in DM.

DM-induced vascular diseases including macrovascular and microvascular complications contribute to diminished quality of life and even death. Common risk factors for DM-related vascular disease include hyperglycemia, insulin resistance, dyslipidemia, inflammation, hypercoagulability, hypertension, and atherosclerosis. All of these factors contribute to endothelial dysfunction and related complications [9]. Endothelial cell injury is the main factor leading to vascular complications in DM [11]. Consequently, comprehensive analysis and improvement in endothelial cell function may inhibit the progression of DM and related complications.

Endothelial microparticles (EMPs) are novel biomarkers facilitating the evaluation of endothelial function in DM-related vascular diseases. EMPs are membrane vesicles derived from activated or apoptotic endothelial cells [12, 13]. The quantity of these membrane vesicles and their subtype profile reflect diverse pathophysiological states. Further, EMPs are garnering increased attention as putative intracellular communication vectors. As illustrated in Table 1 [14], multiple* in vivo* and* in vitro* studies demonstrated that EMPs contribute to coagulation [15, 16], disruption of angiogenesis [6, 17], and cerebral capillary damage [18], among other vascular effects [14, 19]. The plasma level of EMPs is significantly higher in DM patients than in age-matched healthy population [20]. Further, the level of plasma EMPs in DM patients with complications is considerably higher than in DM patients without complications [6, 21, 22]. These studies suggest that EMPs may serve as novel biomarkers for the diagnosis, staging, and progression of DM and related vascular complications.

## 2. Role of EMPs in DM

EMPs account for only 5% to 15% of the total MPs [19, 23]. However, they are valuable biomarkers of endothelial cell function and contribute to the pathological progression of multiple vascular diseases in DM. EMPs contain bioactive macromolecules and act as intercellular signals under pathophysiological conditions. They serve as biological markers of altered vascular endothelium and reflect the pathological progression and diminished endothelial function of blood vessels in DM-related vascular diseases.

### 2.1. EMP Production

Combes et al. first observed the release of EMPs measuring 0.1−1 *μ*m in diameter, as membrane vesicles derived from human umbilical vein endothelial cells (HUVECs)* in vitro* following stimulation by tumor necrosis factor *α* (TNF-*α*) [24]. Subsequently, hyperglycemia, bacterial lipopolysaccharide, thrombin, C-reactive protein, active oxygen cluster, and urea were shown to induce the release of EMPs from endothelial cells [25]. Release of EMPs by apoptotic and activated endothelial cells depends on intracellular calcium elevation and disruption of the asymmetrical distribution of membrane phospholipids, which externalizes phosphatidylserine, and leads to reconstruction of the submembrane skeleton and destabilization of the plasmalemma [26]. Endothelial dysfunction in DM patients increases the plasma level of circulating EMPs resulting in vascular dysfunction [27]. MPs derived from endothelial cells under hyperglycemia induce atherogenesis, angiogenesis, and other complications [9, 28].

### 2.2. Characteristics of EMPs

The subtypes and mechanisms of EMPs distinguish activated endothelial cells from apoptotic endothelial cells. As shown in Table 2 [25], the surface of EMPs released from activated endothelial cells carries higher levels of CD54-intracellular adhesion molecule-1 (ICAM-1), CD62E-E-selectin (E-selectin), and CD106-vascular cell adhesion molecule-1 (VCAM-1). In addition, the EMP surface also expresses low levels of CD31-platelet cell adhesion molecule (PECAM-1), CD105-endothelin (endoglin), and CD144-vascular endothelial cell cadherin (VE-cadherin). Compared with EMPs released from activated endothelial cells, the surfaces of EMPs released from apoptotic endothelial cells express higher levels of CD31-PECAM-1, CD105-endoglin, and CD144-VE-cadherin and lower levels of CD54-ICAM-1, CD62E-E-selectin, and CD106-VCAM-1 [5].

Partial antigen-specific epitopes are expressed in other cells and are not unique to endothelial cells. However, EMPs released under different pathological conditions or in response to different stimuli express different antigen-specific epitopes. Hence, the antigen-specific epitopes are used to distinguish EMPs from other MPs and accurately and specifically reflect the endothelial cell function under different pathophysiological conditions. Hyperglycemia in DM patients significantly elevates the levels of CD31^+^/41a^−^ EMPs, CD31^+^/CD42^−^ EMPs, CD105^+^ EMPs, CD106^+^ EMPs, and CD144^+^ EMPs [6, 20, 29].

The relative proportion of CD62E^+^/CD31^+^ EMPs rather than the absolute quantity distinguishes EMPs released by activated endothelial cells from those derived from apoptotic endothelial cells. A greater than 10% concentration of CD62E^+^/CD31^+^ EMPs indicates that the majority is derived from activated endothelial cells, whereas 1% or less indicates origin from apoptotic endothelial cells [20]. In DM patients, the level of CD62E^+^/CD31^+^ EMPs was less than 1%, indicating that most of them were released from apoptotic rather than activated endothelial cells [20].

### 2.3. Potential Pathogenic Effects of EMPs

As illustrated in Table 1 [14], multiple* in vivo* and* in vitro* studies showed that EMPs contribute to coagulation [15, 16], disruption of angiogenesis [6, 17], and cerebral capillary damage [18], in addition to other vascular effects [14, 19]. These EMPs may have pathogenic effects in vascular thrombosis and angiogenesis, which are essential for the development of DM and its complications.

Procoagulant EMPs initiate and propagate coagulation in DM both* in vitro* and* ex vivo* [30–32]. Procoagulant EMPs are significantly increased in patients with DM, even in well-controlled disease without complications and newly diagnosed type 2 diabetes mellitus (T2DM) [29, 33, 34]. In addition, patients with DM accompanied by hypertension, hyperlipidemia, stable coronary disease, angina with or without symptomatic episodes, myocardial infarction, diabetic retinopathy, and nephropathy have significantly increased levels of procoagulant MPs compared with those without DM-related complications [35]. Therefore, elevated levels of MPs may be associated with increased risk of thromboembolic complications in DM.

Taraboletti et al. demonstrated that although the low concentrations of MPs isolated from HUVECs promote angiogenesis* in vitro*, the high levels of EMPs suppress angiogenesis [36]. Consistent with these results, Mezentsev and colleagues reported that isolated EMPs in pathophysiological concentrations impair angiogenesis* in vitro* by affecting the parameters of capillary-like network [37]. Angiogenesis may be either suppressed as in the late stages of diabetic nephropathy or elevated as in diabetic retinopathy. These conditions are characterized by impaired endothelial function and increased number of circulating EMPs [35]. As discussed above, EMPs exhibit pro- and antiangiogenic features and influence angiogenic activities. The underlying mechanisms, however, are not fully understood [35].

## 3. Role of EMPs in the Differential Diagnosis of DM and Its Complications

The plasma levels of EMPs are significantly elevated in patients diagnosed with DM [20], hypertension [38], hypertriglyceridemia, acute coronary artery syndrome [39], and peripheral vascular diseases [19]. Therefore, assessment of the specific relationship between EMP profile and vascular endothelial injury not only deepens our understanding of the pathological progression of DM but also defines novel biomarkers for the diagnosis of DM and its complications. As illustrated in Table 3, the pathways and mechanisms of EMP production vary markedly in different disease states, suggesting that distinct EMP epitopes facilitate the identification of the disease. Compared with healthy controls, the plasma levels of CD31^+^/41a^−^ EMPs, CD105^+^ EMPs, and CD106^+^ EMPs were significantly upregulated in DM patients [20]. By contrast, the plasma level of CD62E^+^ EMPs in DM patients was only slightly higher, without any statistical significance [20].

### 3.1. DM-Induced Macrovascular and Microvascular Complications

Endothelial dysfunction is a crucial pathogenic mechanism in the progression of DM-related macrovascular and microvascular complications. In fact, EMPs facilitate the differentiation of macrovascular from microvascular complications in clinical practice. The plasma levels of CD31^+^/CD42b^−^ EMPs and CD31^+^/AV^+^ EMPs were significantly higher in DM patients with macrovascular complications compared with patients showing microvascular complications. The study found that CD31^+^/AV^+^ EMPs, duration of DM, and BMI were independent variables predictive of macrovascular complications. Independent variables predictive of microvascular complications included HbA1c and duration of DM. Therefore, altered plasma EMP levels are independent risk factors for T2DM complicated by macrovascular complications. Furthermore, EMP levels show higher prognostic accuracy compared with conventional vascular risk factors and blood glucose levels [40].

### 3.2. DM Complicated with Cardiovascular Diseases

Diabetes is a major risk factor for cardiovascular diseases, which in turn aggravate the incidence and progression of DM. Studies reported that 75% of DM cases are complicated with hypertension [38]. The plasma levels of EMPs were significantly higher in T2DM patients complicated with hypertension compared with those without hypertension [8]. The study found that systolic blood pressure, diastolic blood pressure, and mean arterial pressure were all positively correlated with plasma EMP levels. After controlling for lipid levels and factors related to blood glucose, we found that the EMP level was still positively correlated with systolic blood pressure and mean arterial pressure. Therefore, EMP concentration is a potential risk factor for cardiovascular injury in DM patients, which in turn is closely associated with the incidence of hypertension and arterial wall stiffness [38].

Compared with healthy populations, the prevalence of hypertension and other cardiovascular diseases is significantly higher in DM patients and increases the risk of atherosclerosis [5]. However, not all patients with atherosclerosis manifest clinical symptoms, which may contribute to progression and catastrophic cardiovascular events. The plasma level of CD62^+^ EMPs was significantly lower in T2DM patients complicated with asymptomatic atherosclerosis compared with T2DM patients without atherosclerosis. Multivariate analysis revealed that CD62E^+^ EMPs act as potential prognostic indicators for T2DM complicated with asymptomatic atherosclerosis, providing an early biomarker for treatment. Compared with healthy population, patients with T2DM also exhibited significantly elevated plasma levels of CD144^+^ EMPs. The CD144^+^ EMP level is a stronger risk factor for T2DM complicated with CAD, compared with other conventional risk factors. In addition, the level of plasma CD144^+^ EMPs represents a biomarker for the diagnosis of T2DM complicated with CAD without typical angina. Therefore, plasma CD144^+^ EMPs not only contribute to endothelial dysfunction but also serve as a risk factor for the differential diagnosis and treatment of T2DM complicated with CAD [6].

### 3.3. Diabetic Nephropathy

Diabetic nephropathy is a leading cause of renal failure and almost 30% of T2DM patients suffer from diabetic nephropathy [41]. The plasma level of EMPs was elevated in T2DM patients complicated with diabetic nephropathy compared with T2DM patients without renal complications. Further, the plasma level of EMPs was significantly decreased by sarpogrelate hydrochloride, a blocker of serotonin-induced platelet aggregation [42]. Therefore, plasma EMPs may be potential biomarkers for DM-induced vascular complications, especially diabetic nephropathy.

### 3.4. DM-Induced Retinopathy

Diabetic retinopathy remains the primary cause of blindness in adults [43]. The incidence of DM-induced retinopathy is strongly associated with the activation or apoptosis of retinal nerve and vascular endothelial cells [44]. Changes in plasma and intravitreous MPs have been widely investigated in DM patients with retinal nerve degeneration to detect apoptosis of retinal neurons. A few retina-derived EMPs (PNA^+^ or ILB4^+^ MPs) are absent from the plasma of patients with DM-induced retinopathy. In contrast, the intravitreous level of CD144^+^ EMPs was elevated in DM patients complicated with retinopathy compared with DM patients without retinopathy. Further, platelet-derived MPs account for a larger proportion of the total MPs in DM retinopathy patients, whereas EMPs represent a lower percentage. However, the CD144^+^ EMPs represent the largest MP subtype in the vitreous body of DM patients complicated with retinopathy, and CD144^+^ EMP concentration differs significantly from the plasma levels. Bevacizumab therapy significantly reduces intravitreous CD144^+^ EMP levels in DM patients with retinopathy, a finding that may be used to gauge treatment response and disease progression [45].

### 3.5. DM Complicated with Erectile Dysfunction

The incidence of ED is three times higher in DM patients compared with age-matched healthy adults. The plasma levels of CD62E^+^ EMPs were significantly higher in both DM and non-DM patients diagnosed with ED than in control non-DM population without ED, while CD62E^+^ EMP levels did not differ between ED patients with and without DM. Thus, elevated CD62E^+^ EMPs appear to be specific for ED independent of DM. In contrast, the CD31^+^ EMP level was considerably higher in DM patients complicated with ED than in non-DM patients with ED, although the level in the latter population was still significantly higher than in adults without DM or ED. A high level of CD62E^+^ and CD31^+^ EMPs reflects endothelial cell activation, and a low level suggests apoptosis. The proportion is low (20%) in DM patients complicated with ED, suggesting that DM complicated with ED enhances endothelial cell apoptosis [21, 46].

### 3.6. DM and Metabolic Syndrome

Despite similarities, the EMP profile is different between DM and metabolic syndrome. Compared with non-DM metabolic syndrome, the plasma percentage of combined CD62E^+^ and CD31^+^ EMPs was significantly downregulated in T2DM, suggesting that a majority of plasma EMPs are released from apoptotic endothelial cells rather than activated endothelial cells in T2DM patients. Statistical analysis revealed that T2DM, hs-CRP, OPG, and adiponectin are independent factors influencing the decrease in plasma CD62E^+^ plus CD31^+^ EMPs in T2DM, suggesting a role in accelerated endothelial cell apoptosis [22].

## 4. Changes in MicroRNA Levels within Plasma EMPs of DM Patients

The mechanisms controlling the formation, release, and content of MPs are critical issues. Protein, lipid, and RNA levels within EMPs mediate intercellular signaling under physiological and pathological conditions. Studies have shown that endothelial cells possess significantly different miRNA patterns and seem to selectively pack their EMPs with distinct miRNAs in different pathological states [47, 48]. MiRNAs have been implicated in the epigenetic regulation of key metabolic homeostasis including glucose-induced insulin secretion, lipid metabolism, adipose cell differentiation, and inflammatory and antiangiogenic pathways in T2DM and related complications [49]. Nine miRNAs including miR-126, miR-222, miR-let7d, miR-21, miR-30, miR-92a, miR-139, miR-199a, and miR-26a regulate vascular homeostasis. The miR-126 and miR-26a were significantly reduced in DM patients compared with non-DM patients. Patients with low miR-26a and miR-126 levels were at a higher risk for concomitant CAD. Endothelial cells are the major cell sources of MPs containing miR-126 and miR-26a, respectively. Finally, consistent with our clinical results,* in vitro* studies suggest that hyperglycemia reduces the assembly of miR-126 and miR-26a into EMPs [28, 49]. These findings suggest that miRNA is selectively incorporated into EMPs under certain pathophysiological conditions. Increasing evidence suggests the potential role of EMP-associated miRNA signatures in body fluids or peripheral blood as biomarkers that predict metabolic diseases [35].

## 5. Plasma EMPs Are Altered in Response to Therapy

Plasma EMP levels are altered following treatment with insulin, sulfonylurea, biguanide, or thiazolidinedione. As illustrated in Table 4, pioglitazone effectively reduced the plasma level of CD31^+^ EMPs compared with metformin and significantly improved the risk factors for CAD in T2DM patients. In addition, a decrease in the plasma CD31^+^ EMPs level was strongly correlated with an increase in adiponectin level and decline in C-reactive protein expression in univariate analysis and further correlated with the level of adiponectin and C-reactive protein in multivariate analysis [34].

The levels of plasma EMPs are significantly higher in hypertensive patients with DM than in normotensive and non-DM controls. Although the levels in total cholesterol, triglycerides, hemoglobin A1c, fasting glucose, and body weight did not demonstrate any differences after CR nifedipine administration, the plasma EMPs decreased significantly in hypertensive patients with DM after 3 months of nifedipine CR (controlled release nifedipine: 20 mg/day) treatment [50]. In addition, losartan also significantly decreased the level of plasma EMPs in T2DM patients with hypertension at a dose of 50 mg/d for 24 weeks. Simvastatin significantly decreased the level of plasma EMPs in T2DM patients with hypertension and hyperlipidemia at a dose of 10 mg/d for 24 weeks [51].

The study found that the level of plasma EMPs was decreased significantly in DM patients with hyperlipidemia and high angiopoietin-2 (Ang-2) level (Ang-2 ≥ 3.6 ng/mL) after 6 months of eicosapentaenoic acid (EPA) treatment. Compared with the control group, the level of plasma EMPs in patients with low Ang-2 level (<3.6 ng/mL) after 6 months of EPA treatment and in patients with high Ang-2 level after 3 months of EPA treatment showed no significant difference [52]. In summary, altered plasma EMP levels correspond to changes in pathophysiology following treatment, which suggests that EMPs are potential biomarkers in DM therapy.

## 6. Conclusion

Despite unclear mechanisms and carrier information of EMPs in DM patients, there is no doubt that EMPs act as biomarkers of endothelial cell function during the pathological progression of DM and related vascular diseases. Furthermore, EMPs are promising diagnostic markers of disease progression, treatment, and clinical prognosis of DM and associated vascular illnesses.

## Figures and Tables

**Table 1:** The role plasma EMPs play in DM.

	Animal model	Clinical study	References
EMPs in DM		Increased procoagulant activity (*ex vivo*)	[16]
		Faster brachial-ankle pulse-wave velocity and reduced FMD (*in vivo*)	[29]
		CAD (*in vivo*)	
		Impaired angiogenesis (*ex vivo*)	[6, 17]
		Progression and severity of retinopathy (*in vivo*)	[30]
	Impaired cerebral microvascular density and impaired EPC functions (*in vivo* and *ex vivo*)		[18]

**Table 2:** Antigenic epitope in the surface of EMPs. Many studies have demonstrated that activation and apoptosis endothelial cells release EMPs, whose surface carried different antigen epitope [25].

CD marker	Antigen	Expression	References
CD31	PECAM-1	Apoptosis	[20, 53, 54]
CD51	Integrin av	Apoptosis	[55]
CD54	ICAM-1	Activation	[54]
CD62E	E-selectin	Activation	[20, 54]
CD105	Endoglin	Apoptosis	[20, 54]
CD106	VCAM-1	Activation	[20, 54]
CD144	VE-cadherin	Apoptosis	[20]
CD146	MelCAM	Apoptosis	[12]

CD, cluster of differentiation; EMPs, endothelial microparticles; E-selectin, endothelial-selectin; ICAM-1, intercellular adhesion molecule-1; PECAM-1, platelet endothelial cell adhesion molecule-1; VCAM-1, vascular cell adhesion molecule-1; VE-cadherin, vascular endothelial-cadherin.

**Table 3:** Significance of different antigenic epitope of EMPs in diabetic patients.

Antigen epitope	Disease		References
CD31^+^/41a^−^	DM	CD31^+^/41a^−^ EMPs: 22238 to 157 × 10^4^/*μ*L [DM versus control], *P* = 0.0006	[20]

CD31^+^/CD42^−^	DM	(1) CD31^+^/CD42^−^ EMPs in DM patients were significantly higher than those in healthy controls (*P* < 0.001)	[29]
		(2) Age was positively correlated with CD31^+^/CD42^−^ EMPs (rs = 0.322, *P* = 0.010)	[29]
		(3) The systolic blood pressure was positively correlated with CD31^+^/CD42^−^ EMPs (rs = 0.329, *P* = 0.008)	[29]
		(4) HbA1c level was positively correlated with CD31^+^/CD42^−^ EMPs (rs = 0.337, *P* = 0.008)	[29]
		(5) Fasting blood glucose level was positively correlated with CD31^+^/CD42^−^ EMPs (rs = 0.275, *P* = 0.029)	[29]
		(6) CD31^+^/CD42^−^ EMPs were independently correlated with FMD and baPWV; FMD was negatively correlated with CD31^+^/CD42^−^ EMPs (rs = −0.441, *P* = 0.008); the level of baPWV was correlated with CD31^+^/CD42^−^ EMPs (rs = 0.497, *P* < 0.001)	[29]
	DM macrovascular complications	(7) The CD31^+^/CD42b^−^ EMPs levels were higher in DM patients with macroangiopathy than in DM patients with microangiopathy and no complications	[40]

CD31^+^	T2DM	(1) Compared with metformin, pioglitazone treatment improved the imbalance between endothelial damage and repair capacity and led to more favourable changes in coronary risk factors in patients with newly diagnosed T2DM	[34]
		(2) The univariate analysis showed that the decrease in circulating EMPs was significantly correlated with increase in adiponectin (rs = −0.391, *P* = 0.01) and with decrease in CRP concentrations (rs = 0.416, *P*= 0.01), which remained significant in multivariate analysis (rs = −0.321, 95% CI 0.245 to 0.402, for adiponectin, and rs = 0.265, 95% CI 0.299 to 0.406, for CRP)	[34]
		(3) Levels of EMPs expressing CD31 were significantly different among study groups (*P* < 0.01). DM patients were found to have the highest levels of CD31^+^ EMPs, while non-DM men without ED were those with the lowest levels; non-DM men with ED had CD31^+^ EMPs levels that were intermediate (*P*< 0.05 for both comparisons, DM versus non-DM men with ED, and non-DM men with ED versus non-DM men without ED)	[46]
		(4) Among T2DM patients, an increased level of CD31^+^/annexin V^+^ MPs was significantly associated with asymptomatic atherosclerosis	[56]

CD51^+^	DM	(1) CD51^+^ EMPs in DM patients were significantly higher than those in healthy controls (*P* < 0.001)	[29]
		(2) Age was positively correlated with CD51^+^ EMPs (rs = 0.367, *P* = 0.003)	[29]
		(3) The systolic blood pressure was positively correlated with CD51^+^ EMPs (rs = 0.311, *P* = 0.013)	[29]
		(4) HbA1c level was positively correlated with CD51^+^ EMPs (rs = 0.266, *P* = 0.038)	[29]
		(5) CD51^+^ EMPs were independently correlated with FMD and baPWV; FMD was negatively correlated with CD51^+^ EMPs (rs = −0.405, *P* = 0.016); the level of baPWV was correlated with CD51^+^ EMPs (rs = 0.428, *P* = 0.001)	[29]

CD51^+^/CD41^−^	DM	CD51^+^/CD41^−^ EMPs levels correlated with albuminuria and microvascular complications (e.g., diabetic nephropathy)	[16]

CD62E	DM	(1) Plasma CD62E^+^ EMPs levels were significantly higher when the patient is suffering from erectile dysfunction with or without DM	[46]
	T2DM	(2) Among T2DM patients, decreased CD62E^+^ EMPs were significantly associated with asymptomatic atherosclerosis	[26]

CD105^+^	DM	(1) CD105^+^ EMPs: 2200 to 390 × 10^3^/*μ*L [DM versus control], *P* = 0.002	[20]
	DM	(2) CD105^+^ EMPs may play a critical role in the development and progression of diabetic retinopathy	[57]

CD106^+^	DM	CD106^+^ EMPs: 4939 to 740 × 10^3^/*μ*L [DM versus control], *P* = 0.001	[20]

CD144^+^	DM	(1) CD144^+^ EMPs: 0.541 (0.423–0.652) × 10^6^/mL	[6]
	DM with CAD	(2) Identified a subpopulation of T2DM patients at risk of developing CAD; CD144^+^ EMPs: 0.706 (0.577–1.067) × 10^6^/mL	
	DM without MetS	(3) CD144^+^ EMPs: 139.07 (81.6–271.5) × 10^3^/mL	[17, 58]
	DM with MetS	(4) CD144^+^ EMPs: 251.80 (121.2–499.3) × 10^3^/mL; associated with oxidative stress and MetS and negatively correlated with HDLC levels	
	DM with ACS	(5) CD144^+^EMPs: 442.27 (154.2–826.9) × 10^3^/mL	[6]

CD146	DM with MetS	Associated with the development of MetS	[59]

**Table 4:** The effects of drugs on plasma levels of EMPs in patients with DM.

Pharmacological effects	Drugs	Patient or cells	The effects of drugs on EMPs	References
Antihypertensive	Nifedipine	T2DM with hypertension	Decrease	[50]
	Losartan	T2DM with hypertension	Decrease	[51]

Antiatherosclerosis	Simvastatin	HUVEC	Increase	[60]
	Simvastatin + losartan	T2DM with hyperlipidemia and hypertension	Decrease	[51]
	Eicosapentaenoic acid	T2DM with hyperlipidemia	Decrease	[52]
	Vitamin C	T2DM with acute myocardial infarction	Decrease	[33]

Antihyperglycemic	Pioglitazone	T2DM	Decrease	[34]

## References

[1] Wilkin T. J. (2009). The accelerator hypothesis: a review of the evidence for insulin resistance as the basis for type i as well as type II diabetes. *International Journal of Obesity*.

[2] Wang Y., Wei F., Sun C., Li Q. (2016). The Research of Improved Grey GM (1, 1) model to predict the postprandial glucose in Type 2 diabetes. *BioMed Research International*.

[3] León L. E., Rani S., Fernandez M., Larico M., Calligaris S. D. (2016). Subclinical detection of diabetic cardiomyopathy with microRNAs: challenges and perspectives. *Journal of Diabetes Research*.

[4] Kagota S., Maruyama K., McGuire J. J. (2016). Characterization and functions of protease-activated receptor 2 in obesity, diabetes, and metabolic syndrome: a systematic review. *BioMed Research International*.

[5] Aurelian S. M., Cheţa D. M., Onicescu D. (2014). Microvesicles-potential biomarkers for the interrelations atherosclerosis/type 2 diabetes mellitus. *Romanian Journal of Morphology and Embryology*.

[6] Koga H., Sugiyama S., Kugiyama K. (2005). Elevated levels of VE-cadherin-positive endothelial microparticles in patients with type 2 diabetes mellitus and coronary artery disease. *Journal of the American College of Cardiology*.

[7] Kotani K., Yamada T. (2014). Association between urinary 8-OHdG and pulse wave velocity in hypertensive patients with type 2 diabetes mellitus. *Singapore Medical Journal*.

[8] Nomura S., Inami N., Shouzu A., Urase F., Maeda Y. (2009). Correlation and association between plasma platelet-, monocyte- and endothelial cell-derived microparticles in hypertensive patients with type 2 diabetes mellitus. *Platelets*.

[9] Alexandru N., Badila E., Weiss E., Cochior D., Stępień E., Georgescu A. (2016). Vascular complications in diabetes: microparticles and microparticle associated microRNAs as active players. *Biochemical and Biophysical Research Communications*.

[10] Kozakova M., Palombo C. (2016). Diabetes mellitus, Arterialwall, and cardiovascular risk assessment. *International Journal of Environmental Research and Public Health*.

[11] Kotani K. (2016). Plasma lipoprotein-associated phospholipase A_2_ levels correlated with the cardio-ankle vascular index in long-term type 2 diabetes mellitus patients. *International Journal of Molecular Sciences*.

[12] Mallat Z., Benamer H., Hugel B. (2000). Elevated levels of shed membrane microparticles with procoagulant potential in the peripheral circulating blood of patients with acute coronary syndromes. *Circulation*.

[13] Wan Z., Yu L., Cheng J. (2016). Circulating endothelial microparticles and correlation of serum 1,25-dihydroxyvitamin D with Adiponectin, nonesterified fatty acids, and glycerol from middle-aged men in China. *BioMed Research International*.

[14] Agouni A., Andriantsitohaina R., Martinez M. C. (2014). Microparticles as biomarkers of vascular dysfunction in metabolic syndrome and its individual components. *Current Vascular Pharmacology*.

[15] Dignat-George F., Boulanger C. M. (2011). The many faces of endothelial microparticles. *Arteriosclerosis, Thrombosis, and Vascular Biology*.

[16] Sabatier F., Darmon P., Hugel B. (2002). Type 1 and type 2 diabetic patients display different patterns of cellular microparticles. *Diabetes*.

[17] Bernard S., Loffroy R., Sérusclat A. (2009). Increased levels of endothelial microparticles CD144 (VE-Cadherin) positives in type 2 diabetic patients with coronary noncalcified plaques evaluated by multidetector computed tomography (MDCT). *Atherosclerosis*.

[18] Chen J., Chen S., Chen Y. (2011). Circulating endothelial progenitor cells and cellular membrane microparticles in db/db diabetic mouse: possible implications in cerebral ischemic damage. *American Journal of Physiology—Endocrinology and Metabolism*.

[19] Markiewicz M., Richard E., Marks N., Ludwicka-Bradley A. (2013). Impact of endothelial microparticles on coagulation, inflammation, and angiogenesis in age-related vascular diseases. *Journal of Aging Research*.

[20] Tramontano A. F., Lyubarova R., Tsiakos J., Palaia T., Deleon J. R., Ragolia L. (2010). Circulating endothelial microparticles in diabetes mellitus. *Mediators of Inflammation*.

[21] Esposito K., Ciotola M., Giugliano F. (2007). Endothelial microparticles correlate with erectile dysfunction in diabetic men. *International Journal of Impotence Research*.

[22] Berezin A. E., Kremzer A. A., Samura T. A., Berezina T. A., Kruzliak P. (2015). Impaired immune phenotype of circulating endothelial-derived microparticles in patients with metabolic syndrome and diabetes mellitus. *Journal of Endocrinological Investigation*.

[23] Leroyer A. S., Isobe H., Lesèche G. (2007). Cellular origins and thrombogenic activity of microparticles isolated from human atherosclerotic plaques. *Journal of the American College of Cardiology*.

[24] Combes V., Simon A.-C., Grau G.-E. (1999). In vitro generation of endothelial microparticles and possible prothrombotic activity in patients with lupus anticoagulant. *The Journal of Clinical Investigation*.

[25] Yong P. J. A., Koh C. H., Shim W. S. N. (2013). Endothelial microparticles: missing link in endothelial dysfunction?. *European Journal of Preventive Cardiology*.

[26] Schiro A., Wilkinson F. L., Weston R., Smyth J. V., Serracino-Inglott F., Alexander M. Y. (2014). Endothelial microparticles as conveyors of information in atherosclerotic disease. *Atherosclerosis*.

[27] Leroyer A. S., Tedgui A., Boulanger C. M. (2008). Microparticles and type 2 diabetes. *Diabetes & Metabolism*.

[28] Jansen F., Wang H., Przybilla D. (2016). Vascular endothelial microparticles-incorporated microRNAs are altered in patients with diabetes mellitus. *Cardiovascular Diabetology*.

[29] Feng B., Chen Y., Luo Y., Chen M., Li X., Ni Y. (2010). Circulating level of microparticles and their correlation with arterial elasticity and endothelium-dependent dilation in patients with type 2 diabetes mellitus. *Atherosclerosis*.

[30] Tsimerman G., Roguin A., Bachar A., Melamed E., Brenner B., Aharon A. (2011). Involvement of microparticles in diabetic vascular complications. *Thrombosis and Haemostasis*.

[31] Morel O., Hugel B., Jesel L. (2004). Sustained elevated amounts of circulating procoagulant membrane microparticles and soluble GPV after acute myocardial infarction in diabetes mellitus. *Thrombosis and Haemostasis*.

[32] Tripodi A., Branchi A., Chantarangkul V. (2011). Hypercoagulability in patients with type 2 diabetes mellitus detected by a thrombin generation assay. *Journal of Thrombosis and Thrombolysis*.

[33] Morel O., Jesel L., Hugel B. (2003). Protective effects of vitamin C on endothelium damage and platelet activation during myocardial infarction in patients with sustained generation of circulating microparticles. *Journal of Thrombosis and Haemostasis*.

[34] Esposito K., Maiorino M. I., Di Palo C. (2011). Effects of pioglitazone versus metformin on circulating endothelial microparticles and progenitor cells in patients with newly diagnosed type 2 diabetes-a randomized controlled trial. *Diabetes, Obesity and Metabolism*.

[35] Wang Y., Chen L.-M., Liu M.-L. (2014). Microvesicles and diabetic complications—novel mediators, potential biomarkers and therapeutic targets. *Acta Pharmacologica Sinica*.

[36] Taraboletti G., D'Ascenzo S., Borsotti P., Giavazzi R., Pavan A., Dolo V. (2002). Shedding of the matrix metalloproteinases MMP-2, MMP-9, and MT1-MMP as membrane vesicle-associated components by endothelial cells. *American Journal of Pathology*.

[37] Mezentsev A., Merks R. M. H., O'Riordan E. (2005). Endothelial microparticles affect angiogenesis in vitro: role of oxidative stress. *American Journal of Physiology—Heart and Circulatory Physiology*.

[38] Chen Y., Feng B., Li X., Ni Y., Luo Y. (2012). Plasma endothelial microparticles and their correlation with the presence of hypertension and arterial stiffness in patients with type 2 diabetes. *Journal of Clinical Hypertension*.

[39] Bernal-Mizrachi L., Jy W., Fierro C. (2004). Endothelial microparticles correlate with high-risk angiographic lesions in acute coronary syndromes. *International Journal of Cardiology*.

[40] Jung K.-H., Chu K., Lee S.-T. (2011). Risk of macrovascular complications in type 2 diabetes mellitus: endothelial microparticle profiles. *Cerebrovascular Diseases*.

[41] Shi J., Jiang S., Qiu D. (2016). Rapid identification of potential drugs for diabetic nephropathy using whole-genome expression profiles of glomeruli. *BioMed Research International*.

[42] Williams M. S., Rogers H. L., Wang N.-Y., Ziegelstein R. C. (2014). Do platelet-derived microparticles play a role in depression, inflammation, and acute coronary syndrome?. *Psychosomatics*.

[43] Nawaz M. I., Abouammoh M., Khan H. A., Alhomida A. S., Alfaran M. F., Ola M. S. (2013). Novel drugs and their targets in the potential treatment of diabetic retinopathy. *Medical Science Monitor*.

[44] Al-Shabrawey M., Zhang W., McDonald D. (2015). Diabetic retinopathy: mechanism, diagnosis, prevention, and treatment. *BioMed Research International*.

[45] Chahed S., Leroyer A. S., Benzerroug M. (2010). Increased vitreous shedding of microparticles in proliferative diabetic retinopathy stimulates endothelial proliferation. *Diabetes*.

[46] Esposito K., Ciotola M., Giugliano F. (2008). Phenotypic assessment of endothelial microparticles in diabetic and nondiabetic men with erectile dysfunction. *Journal of Sexual Medicine*.

[47] Diehl P., Fricke A., Sander L. (2012). Microparticles: major transport vehicles for distinct microRNAs in circulation. *Cardiovascular Research*.

[48] Müller G. (2012). Microvesicles/exosomes as potential novel biomarkers of metabolic diseases. *Diabetes, Metabolic Syndrome and Obesity: Targets and Therapy*.

[49] Zampetaki A., Kiechl S., Drozdov I. (2010). Plasma MicroRNA profiling reveals loss of endothelial MiR-126 and other MicroRNAs in type 2 diabetes. *Circulation Research*.

[50] Nomura S., Inami N., Kimura Y. (2007). Effect of nifedipine on adiponectin in hypertensive patients with type 2 diabetes mellitus. *Journal of Human Hypertension*.

[51] Nomura S., Shouzu A., Omoto S., Nishikawa M., Fukuhara S., Iwasaka T. (2004). Losartan and simvastatin inhibit platelet activation in hypertensive patients. *Journal of Thrombosis and Thrombolysis*.

[52] Nomura S., Shouzu A., Omoto S. (2009). Effects of eicosapentaenoic acid on endothelial cell-derived microparticles, angiopoietins and adiponectin in patients with type 2 diabetes. *Journal of Atherosclerosis and Thrombosis*.

[53] Wang J.-M., Wang Y., Huang J.-Y. (2007). C-reactive protein-induced endothelial microparticle generation in HUVECs is related to BH4-dependent NO formation. *Journal of Vascular Research*.

[54] van Ierssel S. H., Van Craenenbroeck E. M., Conraads V. M. (2010). Flow cytometric detection of endothelial microparticles (EMP): effects of centrifugation and storage alter with the phenotype studied. *Thrombosis Research*.

[55] Tramontano A. F., O'Leary J., Black A. D., Muniyappa R., Cutaia M. V., El-Sherif N. (2004). Statin decreases endothelial microparticle release from human coronary artery endothelial cells: implication for the Rho-kinase pathway. *Biochemical and Biophysical Research Communications*.

[56] Berezin A. E., Kremzer A. A., Berezina T. A., Martovitskaya Y. V. (2016). The pattern of circulating microparticles in patients with diabetes mellitus with asymptomatic atherosclerosis. *Acta Clinica Belgica*.

[57] Malik R. A., Li C., Aziz W. (2005). Elevated plasma CD105 and vitreous VEGF levels in diabetic retinopathy. *Journal of Cellular and Molecular Medicine*.

[58] Helal O., Defoort C., Robert S. (2011). Increased levels of microparticles originating from endothelial cells, platelets and erythrocytes in subjects with metabolic syndrome: relationship with oxidative stress. *Nutrition, Metabolism and Cardiovascular Diseases*.

[59] Agouni A., Lagrue-Lak-Hal A. H., Ducluzeau P. H. (2008). Endothelial dysfunction caused by circulating microparticles from patients with metabolic syndrome. *American Journal of Pathology*.

[60] Diamant M., Tushuizen M. E., Abid-Hussein M. N. (2008). Simvastatin-induced endothelial cell detachment and microparticle release are prenylation dependent. *Thrombosis and Haemostasis*.

